# Marine Compounds and Cancer: Updates 2022

**DOI:** 10.3390/md20120759

**Published:** 2022-12-01

**Authors:** Sergey A. Dyshlovoy, Friedemann Honecker

**Affiliations:** 1 Department of Oncology, University Cancer Center Hamburg, University Medical Center Hamburg-Eppendorf, 20246 Hamburg, Germany; 2 Laboratory of Pharmacology, A.V. Zhirmunsky National Scientific Center of Marine Biology, Far Eastern Branch, Russian Academy of Sciences, 690041 Vladivostok, Russia; 3 Martini-Klinik Prostate Cancer Center, University Medical Center Hamburg-Eppendorf, 20246 Hamburg, Germany; 4 Tumor- und BrustZentrum Ostschweiz, 9016 St. Gallen, Switzerland

The field of marine bioactive compounds (marine drugs) has evolved significantly in recent years. By the end of 2022, we will have seventeen marine-derived drugs approved for clinical use. Twelve of them (71%) are approved for the treatment of various types of cancer. This fact underlines the antitumor efficacy of many molecules produced by marine organisms. The high antineoplastic activity of several of these metabolites is regularly reported in the literature. Biological effects can often be explained by their unique chemical structure and primary natural defensive or allelochemical function, i.e., the impact of one species on the survival and growth of other species by producing specific mediators.

The field of marine anticancer compounds is currently growing exponentially (Figure 1). Since 2020, when we published our previous editorial [1], two new marine-derived anticancer medications have been approved. Both drugs are so-called antibody-drug conjugates (ADC) that are constructed using an antibody specific to a cell surface protein overexpressed by cancer cells and a cytotoxic “warhead” represented in both cases by monomethyl auristatin E (MMAE), which acts via inhibition of tubulin polymerization [2]. MMAE is a synthetic derivative of the tetrapeptide dolastatin-10, which was initially isolated from *Dolabella auricularia* but is produced by symbiotic cyanobacteria [3,4]. Six ADC drugs now possess monomethyl auristatin E or F (MMAE or MMAF) as a cytotoxic moiety (Figure 1). Moreover, many more related ADCs are at various stages of clinical trials [5] and, therefore, might be expected to enter the clinical routine before too long.

Thus, the complete list of the twelve currently approved marine-derived anticancer drugs, which is represented in Figure 1, is as follows: 

Cytarabine (Cytosar-U^®^), Trabectidine (Yondelis^®^), Eribulin mesylate (Halaven^®^), Brentuximab vedotin (Adcetris^®^), Panobinostat (Farydak^®^), Plitidepsin (Aplidin^®^), Polatuzumab vedotin (Polivy^TM^), Enfortumab vedotin (PADCEV^TM^), Belantamab mafodotin (Blenrep^TM^), Lurbinectedin (Zepzelca^TM^), Tisotumab vedotin-tftv (TIVDAK^TM^), and Disitamab vedotin (Aidixi™). The first ten of this list have been previously reviewed by us and others elsewhere [1,2,6,7,8,9]. The two new drugs approved in the period from 2020 until the end of 2022 are

- **Tisotumab vedotin-tftv** (TIVDAK^TM^, produced and sold by Seagen), an ADC consisting of MMAE conjugated with a monoclonal antibody tisotumab specific to tissue factor (factor III, CD142). The FDA approved the drug in 2021 to treat metastatic cervical cancer [10].
- **Disitamab vedotin** (RC-48, Aidixi™, produced and sold by Remegen Biosciences), an ADC consisting of MMAE conjugated with a monoclonal antibody disitamab specific to HER2. The Chinese Center for Drug Evaluation (CDE) approved the drug in China in 2021 for the treatment of HER2-expressing gastric cancer and is currently undergoing clinical trials in the US [11].

A paramount contribution to the systemic review of recent progress and updates on marine-derived drug discovery and development has been made by Prof. Alejandro M. S. Mayer and his team. They maintain and support the Marine Pharmacology web page (https://www.marinepharmacology.org, accessed on 24 November 2022) and regularly publish critical reviews on marine pharmacology, which we highly recommend reading [12,13,14,15]. 

To keep track of this dynamic area, as well as to offer a platform to publish and share the recent results, we started the Topical Collection “Marine Compounds and Cancer” (http://www.mdpi.com/journal/marinedrugs/special_issues/marine-compounds-cancer, accessed on 24 November 2022) in 2015 [6]. Many authors have contributed to this Topical Collection in the last two years, which we would like to acknowledge here. 

The group of Fagon reported the chemopreventive activity of the phlorotannin-rich fraction of the brown algae *Ascophyllum nodosum* and *Fucus vesiculosus* in the models of benzo[a]pyrene-induced carcinoma in vitro. The mechanism of this activity was identified as the inhibition of carcinogen-induced activation of P2X7 [16]. Hernández-Balmaseda and colleagues showed the ability of extracts of the marine alga *Thalassia testudinum* to suppress the growth and progression of colon cancer in vivo. The mechanisms of this effect were identified as the inhibition of angiogenesis, induction of autophagy, and stimulation of anticancer immunity [17]. Zhang et al. reported the anticancer and antimetastatic activity of the marine compound penisuloxazin A in a breast cancer model in vitro. The mechanism of this effect was described as C-terminal inhibition of the heat shock protein Hsp90 and turnover of epithelial-mesenchymal transition (EMT) [18]. Spirin and colleagues described the effect of the synthetic marine alkaloid 3,10-dibromofascaplysin on leukemia cells. The authors found that this alkaloid can induce apoptosis and activate an E2F1 transcriptional factor. Additionally, a synergistic effect of 3,10-dibromofascaplysin in combination with cytarabine was reported [19]. A review article by Dyshlovoy represents an overview of the literature published within the last 8 years and is mainly devoted to marine cancer-preventive compounds [20]. The group of Li studied the activity of actinomycin V in human colorectal carcinoma models. The authors reported the anticancer activity of this marine compound that is executed via targeting mitochondrial apoptotic and PI3K/AKT pathways [21]. Bjerknes and colleagues characterized a salmon protein hydrolysate and showed its synergistic effect in combination with bicalutamide in human prostate cancer cells. The mechanism of this phenomenon was described as the modulation of iron homeostasis [22]. The group of von Amsberg investigated the cytotoxic anticancer activity of N-methylpretrichodermamide B in drug-resistant prostate cancer cells in vitro. The authors reported that the compound showed no cross-resistance with docetaxel. Additionally, it was demonstrated that N-methylpretrichodermamide B could synergize with docetaxel, which was explained by p-glycoprotein inhibitory activity [23]. Finally, Tiasto et al. reported an isolation of κ- and λ-carrageenans from the marine alga *Chondrus armatus*. They characterized its anticancer activity and partial mode of action using several models of human gastrointestinal [24].

With this, we would like to thank all the colleagues who have chosen our Topical Collection “Marine Compounds and Cancer” as a platform for publishing their research! 

## Figures and Tables

**Figure 1:**
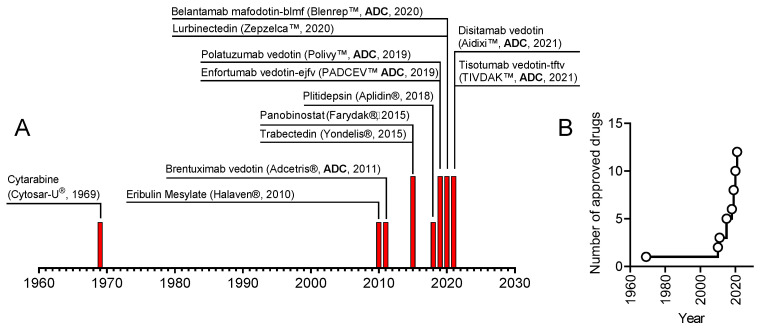
(**A**) The timeline represents marine-derived anticancer drugs approved in the indicated year. (**B**) The total number of marine-derived anticancer drugs approved.
